# Role and Mechanism of *BRIP1* in Anoikis Resistance of Gastric Cancer

**DOI:** 10.3390/ijms27052409

**Published:** 2026-03-05

**Authors:** Shijiao Zhang, Ai Chen, Liyang Chen, Chuanli Yang, Yan Shen, Xiaobing Shen

**Affiliations:** 1 Key Laboratory of Environmental Medicine Engineering of Ministry of Education, School of Public Health, Southeast University, Nanjing 210009, China; 2 School of Medical, Southeast University, Nanjing 210009, China

**Keywords:** gastric cancer, anoikis, BRIP1, ROS, PI3K/Akt signaling pathway

## Abstract

To assess the therapeutic relevance of *BRIP1* in gastric cancer (GC), we examine its functional role in conferring resistance to anoikis within GC cells and elucidate the oncogenic signaling pathways modulated by *BRIP1*. By integrating the Cancer Genome Atlas (TCGA) and Gene Set Enrichment Analysis (GSEA) databases with Least Absolute Shrinkage and Selection Operator (LASSO) regression, a novel risk score to stratify GC patients based on prognosis was generated, and a significantly differentially expressed gene, *BRIP1*, was validated through reverse transcription quantitative polymerase chain reaction (RT-qPCR). Protein expression associated with apoptosis, cell cycle, and epithelial-mesenchymal transformation (EMT) was quantified via RT-qPCR and Western blot (WB). 5-Ethynyl-2′-deoxyuridine (EdU) and cell counting kit-8 (CCK-8) assays were conducted to quantify proliferative activity. The protein level in axillary tumor tissues of nude mice was detected by immunohistochemistry (IHC). We established an eight-gene anoikis-related prognostic risk assessment model (*DUSP1*, *VCAN*, *P3H2*, *TXNIP*, *BRIP1*, *FGD6*, *GPX3*, and *RLN2*) for GC. Multivariate Cox regression confirmed the risk score as an independent prognostic factor. Among these genes, *BRIP1* showed significant differential expression between tumor and normal tissues, as well as normal gastric mucosal epithelial cells and GC cells. Mechanistically, *BRIP1* conferred anoikis resistance to GC cells by suppressing the generation of reactive oxygen species (ROS). We found that the PI3K inhibitor LY294002 counteracted *BRIP1*-driven oncogenic effects, which was evidenced by restored expression of key regulators governing apoptosis, cell cycle progression, and EMT, in addition to suppressed proliferation in GC cells. *BRIP1* is postulated to function upstream of the PI3K/Akt signaling cascade. This study establishes a risk scoring model and identifies *BRIP1* as a potential prognostic marker for GC.

## 1. Introduction

As a major global health burden, gastric cancer (GC) is correlated with considerable morbidity and mortality [1]. Epidemiological data from 2020 estimated approximately 968,000 new cases of GC globally, alongside about 660,000 deaths [2]. Studies have shown that GC is characterized by an unfavorable prognosis, reflected in its elevated mortality and a five-year survival rate below 10% [3]. Therefore, we need to explore effective ways to treat GC.

Anoikis occurs when cells detach from their surrounding extracellular matrix (ECM), leading to their programmed self-destruction [4,5], and it is an important mechanism of host clearance of circulatory tumor cells [6]. Anoikis induces apoptosis in shed cells, thereby inhibiting their reattachment to unsuitable ECM locations, which could otherwise lead to dysplastic growth and neoplasm formation [7]. Compared with normal epithelial cells, tumor cells are not sensitive to anoikis and, therefore, do not need to adhere to ECM to survive and proliferate [6]. Studies have shown that tumors have anoikis resistance, a capability linked to the loss of internal environmental stability, tumor progression, and the formation of distant metastases [8].

Anoikis-related genes (ARGs) can realize the anoikis resistance of tumor cells through epithelial–mesenchymal transformation (EMT) [9] and thus regulate the occurrence and development of tumors. EMT represents a plasticity program through which epithelial cells undergo a specific sequence of changes, resulting in enhanced migratory and invasive capabilities reminiscent of stromal cells [10]. Evidence indicates that EMT underpins key stages of tumor progression, including initiation, invasion, and metastasis [11], and in addition, EMT enables tumor cells to separate from neighboring cells, thereby acquiring the ability of anoikis resistance [12]. It primarily expresses key mesenchymal biomarkers, including *Vimentin*, *α-SMA*, *N-cadherin*, and *Snail* activation [13]. Moreover, many central regulators of EMT exert dual control over tumor progression by coordinately suppressing apoptosis through *Bcl2* upregulation and a concurrent reduction in pro-apoptotic mediators like Caspase-3 and Bax. For instance, Tan et al. demonstrated that *FZD7* can upregulate EMT-related genes and *Bcl2* expression through the downstream key effector *TWIST*, promoting anoikis resistance in ovarian cancer cells and leading to reduced patient survival [14].

Additionally, several ARGs function as mediators of anoikis resistance, enabling tumor cell survival through specific mechanistic adaptations. Reactive oxygen species (ROS) are reactive metabolic byproducts that accumulate under conditions of oxidative stress, which can promote DNA damage and thus lead to potentially carcinogenic mutations [15]. Therefore, ROS are well-established pathogenic contributors to malignant progression, with documented significance in primary tumor growth and metastatic reactivation, and are also necessary for the continuation of the tumor cell cycle [16]. Once ROS levels are elevated, non-ligands of growth factor receptors are activated, and downregulation of pro-apoptotic factors is mediated by redox, further increasing ROS production and resistance to anoikis [17]. ROS can also mediate the occurrence of anoikis resistance in tumor cells by activating signaling pathways [18]. The activated signaling pathways function as key tumor drivers by altering the expression of survival proteins and invasion-promoting factors, a process that subsequently enables evasion of detachment-induced cell death [19]. For example, miR-26a can enhance the apoptotic sensitivity of liver cancer by targeting integrin ITGA5 and negatively regulate the PI3K/Akt pathway to inhibit anoikis resistance of liver cancer [11]. In colorectal cancer, anoikis resistance and cell invasion can be regulated through certain signaling pathways [20].

Anoikis resistance is a key feature of malignant progression in tumor cells [21]. While anoikis resistance in GC cells has garnered significant scientific interest, a critical knowledge gap persists regarding its regulatory mechanisms, rendering this field ripe for systematic investigation.

Consequently, we integrated data from the Cancer Genome Atlas (TCGA) and Gene Set Enrichment Analysis (GSEA) databases to identify critical ARGs in GC. A prognostic risk score model was subsequently established using the Least Absolute Shrinkage and Selection Operator (LASSO) regression algorithm. Among these genes, *BRIP1* was selected as the most promising candidate based on evidence in the literature and was further validated via reverse transcription quantitative polymerase chain reaction (RT-qPCR). We then systematically investigated the role of *BRIP1* in regulating anoikis resistance, proliferation, cell cycle progression, EMT, and in vivo tumorigenesis using a series of molecular biology experiments. These studies were conducted to elucidate the molecular mechanisms governing anoikis resistance in GC cells and to clarify its significance in GC pathogenesis. This work aims to evaluate the potential of *BRIP1* as a target and to provide a theoretical foundation for the identification of novel prognostic biomarkers and treatment strategies in GC.

## 2. Results

### 2.1. BRIP1, an Anoikis-Related Gene, Is Highly Expressed in GC

**Figure 1:**
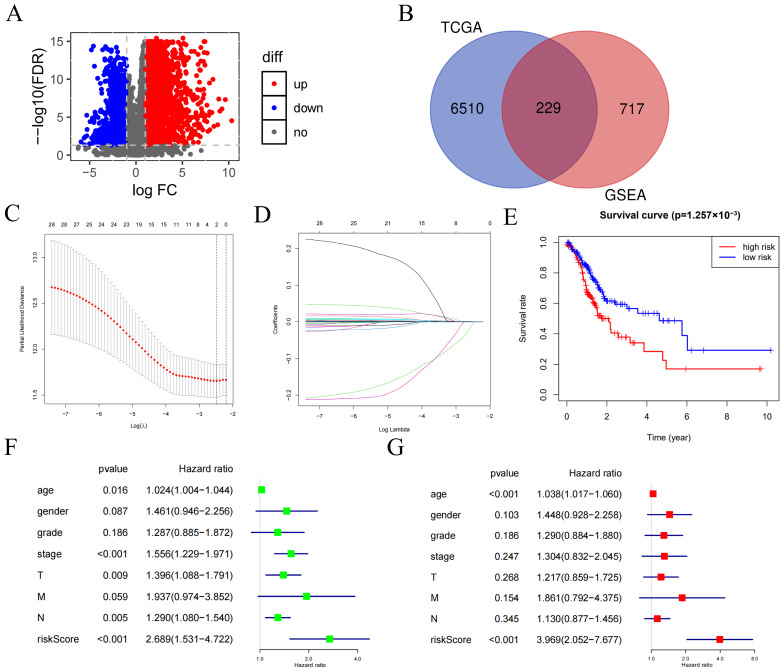
**BRIP1, an anoikis-related gene, is highly expressed in GC.** (**A**) Volcano plot displays DEGs between 375 GC tissues and 32 normal tissues from the TCGA-STAD cohort. (**B**) The intersection of TCGA and GSEA dataset filter ARGs. (**C**,**D**) Prognosis model constructed by LASSO algorithm. (**E**) Patients were stratified into high- (above median) and low-risk (below median) groups. Kaplan–Meier survival curve validates the model. (High-risk group is shown in red, and low-risk group is shown in blue.) (**F**) Univariate Cox regression analysis. (**G**) Multivariate Cox regression analysis. Interpretation of green and red squares: Hazard ratio (HR) > 1 (right of line): Risk factor. HR < 1 (left of line): Protective factor. HR crossing or near 1 (on the line): Non-significant.

Analysis of GC transcriptomic data from TCGA revealed 6739 differentially expressed genes (DEGs). In total, 229 key ARGs were derived by intersecting with 946 ARGs in the GSEA database (Figure 1A,B). Univariate Cox regression (*p* < 0.05) pinpointed 28 significant ARGs. Further, a prognostic model with eight ARGs (*DUSP1*, *VCAN*, *P3H2*, *TXNIP*, *BRIP1*, *FGD6*, *GPX3*, and *RLN2*) was constructed using the LASSO algorithm (Figure 1C,D). The risk score for each patient was calculated as a linear combination of the expression values of eight genes, weighted by their respective coefficients derived from the LASSO regression analysis. The formula was defined as follows: risk score = (0.0014 × *DUSP1*) + (0.0020 × *VCAN*) + (0.0073 × *P3H2*) + (0.0003 × *TXNIP*) + (−0.0617 × *BRIP1*) + (0.0015 × *FGD6*) + (0.0002 × *GPX3*) + (−0.0356 × *RLN2*).

**Figure 2:**
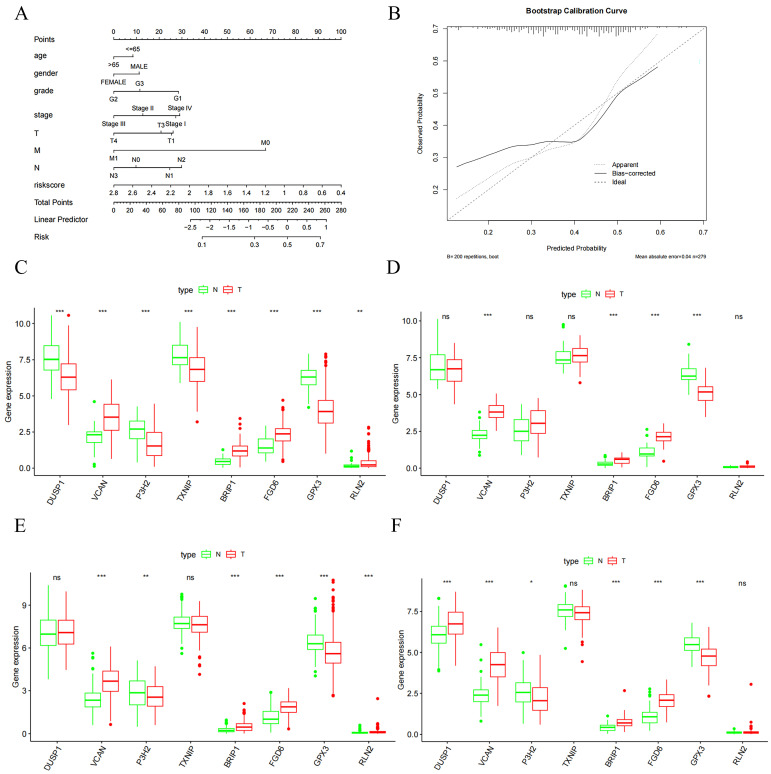
**Categorical mapping construction and database validation.** (**A**,**B**) Nomogram (**A**) and calibration curve (**B**) for predicting patient survival. (**C**–**F**) TCGA (**C**) and GEO datasets GSE179252 (**D**), GSE184336 (**E**), GSE122401 (**F**) verified the DEGs. “ns, not significant; * *p* < 0.05; ** *p* < 0.01; *** *p* < 0.001”.

Using the median risk score (1.1260) as a cutoff, we separated patients into high- and low-risk groups. The high-risk group exhibited significantly poorer overall survival in the Kaplan–Meier analysis (*p* < 0.05, Figure 1E). The prognostic independence of the risk score was confirmed by univariate (Figure 1F) and multivariate Cox regression (Figure 1G). Moreover, the nomogram and its accompanying calibration curve demonstrated favorable predictive accuracy for patient survival (Figure 2A,B).

Validation in TCGA and the Gene Expression Omnibus (GEO) datasets revealed that *VCAN*, *BRIP1*, *FGD6*, and *RLN2* were robustly upregulated in GC tissues. Conversely, *TXNIP*, *P3H2*, *GPX3*, and *DUSP1* were significantly downregulated compared to normal controls (Figure 2C,F). RT-qPCR outcomes verified that *VCAN*, *BRIP1*, *FGD6* and *RLN24* genes were increased in GC tissues, while *TXNIP*, *P3H2*, *GPX3* and *DUSP1* genes were decreased (Scheme S1).

### 2.2. BRIP1 Promotes Anoikis Resistance in GC Cells

**Figure 3:**
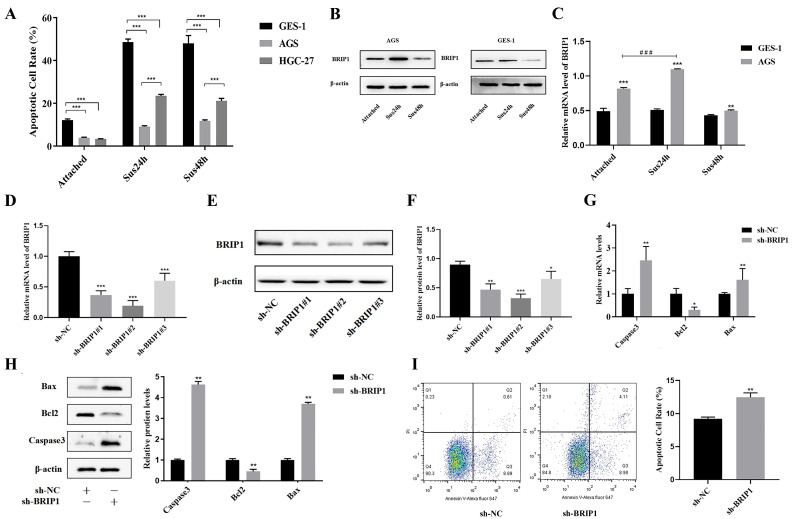
**BRIP1 promotes anoikis resistance in GC cells.** (**A**) Apoptotic rates were assessed in GES-1, AGS and HGC-27 after 24 and 48 h of adherent or suspension culture. (**B**,**C**) The changes in *BRIP1* protein (**B**) and mRNA expression (**C**) in GES-1 and AGS were analyzed after adhesion and suspension culture (24 h, 48 h). (**D**–**F**) Verified efficiency of downregulated *BRIP1*. (**G**) Analysis of the mRNA expression of apoptosis-related genes. (**H**) The expression of apoptosis-related proteins was assessed. (**I**) Analysis of the effect of BRIP1 on apoptosis rate. * *p* < 0.05; ** *p* < 0.01; *** *p* < 0.001. ### *p* < 0.001.

To investigate anoikis resistance in GC, we selected the normal gastric mucosal epithelial cell line GES-1 and two GC cell lines (AGS, HGC-27). Cells were subjected to adherent or suspended culture conditions for 24 and 48 h to induce anoikis. Flow cytometric analysis revealed that suspended GES-1 cells exhibited a significantly higher apoptotic rate than GC cells (*p* < 0.05, Figure 3A, Scheme S2A). Between AGS and HGC-27, AGS cells demonstrated a greater anoikis resistance capacity compared to HGC-27 cells (*p* < 0.05) and were, therefore, chosen for subsequent modeling.

We next assessed *BRIP1* expression under these conditions via RT-qPCR and WB. A marked increase in BRIP1 levels was observed in AGS relative to the GES-1 cell line (*p* < 0.05, Figure 3B,C). Notably, in AGS cells, BRIP1 expression increased significantly after 24 h of suspension culture but decreased after 48 h, suggesting its involvement in the early regulatory response to anoikis and identifying 24 h of suspension as the optimal condition for model establishment.

Following viral transfection, the efficiency of *BRIP1* knockdown and overexpression was validated. The *sh-BRIP1#2* construct showed the most potent knockdown effect (*p* < 0.05, Figure 3D,F), while a pronounced upregulation of *BRIP1* was achieved in the overexpression group (*p* < 0.05, Scheme S2B–D). Stable transmutation strains with downregulated and upregulated *BRIP1* were successfully constructed. Functional experiments demonstrate that knocking down *BRIP1* upregulated Caspase3 and Bax expression and downregulated Bcl2 expression (*p* < 0.05, Figure 3G,H), and the apoptosis rate increased (*p* < 0.05, Figure 3I); *BRIP1* overexpression revealed the opposite trend (*p* < 0.05, Scheme S2E–G). These findings imply that BRIP1 promotes anoikis resistance in GC cells.

### 2.3. BRIP1 Enhances the Proliferative Ability of GC Cells

**Figure 4:**
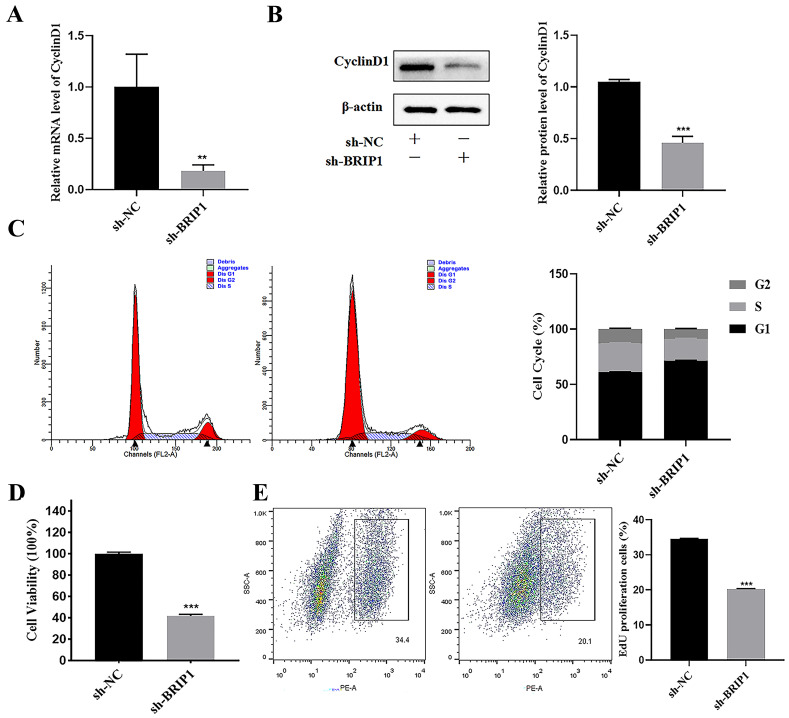
**BRIP1 limits the progression of GC cell cycle and increases the proliferation ability of GC cells.** (**A**) *BRIP1* regulated the expression of *CyclinD1* mRNA. (**B**) WB analysis of CyclinD1 protein expression regulated by *BRIP1*. (**C**) Cell cycle distribution analyzed by flow cytometry following *BRIP1* modulation. The first peak (tallest) represents the G1 phase, the second peak (shorter) represents the G2 phase, and the region between the two peaks represents the S phase. (**D**) Assessment of cell viability by CCK-8 assay. (**E**) Cell proliferation assessed by EdU assay. All experiments were performed on AGS cells. ** *p* < 0.01; *** *p* < 0.001.

To explore the impact of BRIP1 on cell cycle regulation and proliferation in GC progression, the following experiments were conducted: RT-qPCR and WB revealed that knockdown of *BRIP1* significantly decreased the expression of CyclinD1 (*p* < 0.05, Figure 4A,B), whereas its overexpression was the opposite (*p* < 0.05, Scheme S3A,B). Consistent with these findings, flow cytometry revealed that *BRIP1* depletion induced G1 phase arrest, while *BRIP1* overexpression led to S phase accumulation (*p* < 0.05, Figure 4C, Scheme S3C).

We further evaluated proliferative capacity using functional assays. The CCK-8 assay demonstrated that *BRIP1* knockdown inhibited cell proliferation, and its overexpression enhanced it (*p* < 0.05, Figure 4D, Scheme S3D). These results were corroborated by EdU assays, which indicated that DNA replication activity was decreased following *BRIP1* silencing and increased upon *BRIP1* overexpression (*p* < 0.05, Figure 4E, Scheme S3E,F). The above results reflect that BRIP1 could affect the cell cycle process, knockdown of *BRIP1* leads to G1 phase block, and overexpression of *BRIP1* leads to S phase block and significantly affects the proliferation ability of GC.

### 2.4. BRIP1 Promotes EMT in GC and Influences Axillary Tumor Formation in Nude Mice

**Figure 5:**
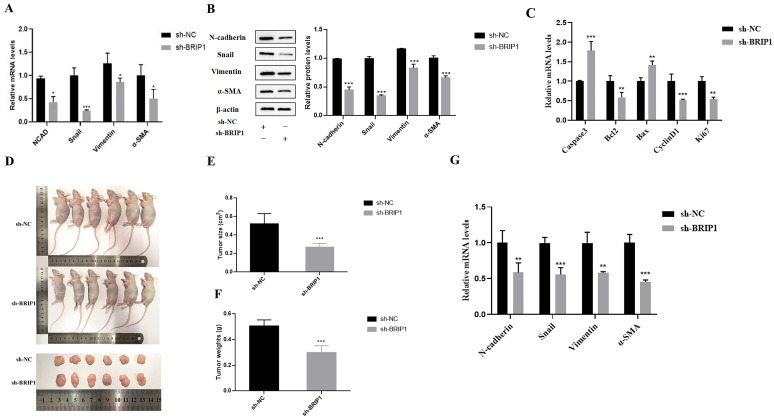
BRIP1 promotes the EMT process of GC, improves the tumor-forming ability of GC cells in vivo, and regulates transcription expression of related genes in tumor tissues. (**A**) The mRNA expression of EMT gene regulated by *BRIP1* was detected. (**B**) Analysis of the expression of EMT protein regulated by *BRIP1*. (**C**) Assessment of the expression of apoptosis, cycle and proliferation genes in tumor tissues. (**D**) In vivo tumor formation experiment with knockdown of *BRIP1*. (**E**,**F**) Tumor size and weight in the *BRIP1* knockdown group. (**G**) Expression of EMT-related genes in tumor tissues. * *p* < 0.05; ** *p* < 0.01; *** *p* < 0.001.

We aimed to elucidate whether BRIP1-mediated anoikis resistance influences the EMT process and in vivo tumorigenicity in GC. The RT-qPCR and WB results indicated that knockdown of *BRIP1* significantly decreased the expression of EMT markers (*N-cadherin*, *Snail*, *Vimentin*, *α-SMA*) (*p* < 0.05, Figure 5A,B), while overexpression of *BRIP1* significantly increased the expression of these markers (*p* < 0.05, Scheme S4A,B). In a nude mouse xenograft model, tumor volume and weight were significantly reduced upon *BRIP1* knockdown and increased upon its overexpression (*p* < 0.05, Figure 5D–F, Scheme S4D–F). Further genetic analysis of tumor tissue demonstrated that *BRIP1* knockdown led to downregulation of *Bcl2*, *CyclinD1*, *Ki67* and EMT-related genes (*p* < 0.05, Figure 5C,G), along with upregulation of *Caspase3* and *Bax* (*p* < 0.05, Figure 5C). Overexpressed *BRIP1* revealed the opposite trend (*p* < 0.05, Scheme S4C,G).

The immunohistochemistry (IHC) of xenograft tissues corroborated these findings at the protein level, showing that *BRIP1* silencing increased Caspase-3 and Bax expression while decreasing Bcl2, Cyclin D1, Ki67, and EMT markers (*p* < 0.05, Scheme S5A,C). Again, *BRIP1* overexpression produced the converse effects (*p* < 0.05, Scheme S5B,D). Collectively, these data confirm that BRIP1 enhances the metastatic potential of GC by driving EMT and significantly promotes tumor growth in vivo through coordinated regulation of apoptosis, cell cycle, proliferation, and EMT processes.

### 2.5. BRIP1 Enhances Malignant Progression of GC by Suppressing ROS Production and Activating the PI3K/Akt Signaling Pathway

**Figure 6:**
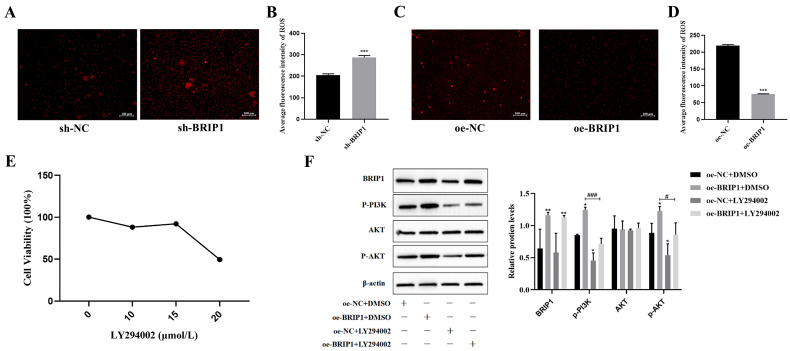
**BRIP1 confers anoikis resistance in GC via inhibition of ROS production and activation of the PI3K/Akt pathway.** (**A**,**B**) Representative fluorescence microscopy images (**A**) and quantitative flow cytometry analysis (**B**) of intracellular ROS levels in AGS cells after *BRIP1* knockdown. The red fluorescence typically represents an elevated level of superoxide anions, suggesting that *BRIP1* knockdown may induce an oxidative stress response in the cells. (**C**,**D**) The effects of overexpression of *BRIP1* on ROS levels were detected. (**E**) Cell viability assessment of LY294002 following several treatment concentrations. (**F**) Relative protein level assessment of BRIP1, p-P13K, AKT, p-AKT following treatments, including the negative control (oe-NC + DMSO), overexpressed *BRIP1* (oe-BRIP1 + DMSO), LY294002 (oe-NC + LY294002), and overexpressed *BRIP1* and LY294002 combination (oe-BRIP1 + LY294002). All experiments were performed on AGS cells. * *p* < 0.05; ** *p* < 0.01; *** *p* < 0.001 versus control; # *p* < 0.05; ### *p* < 0.001.

To explore the underlying mechanism, we first assessed the effect of BRIP1 on intracellular ROS levels. DCFH-DA staining coupled with flow cytometry showed that *BRIP1* knockdown significantly increased ROS production in AGS cells (*p* < 0.05, Figure 6A,B), whereas its overexpression reduced ROS levels (*p* < 0.05, Figure 6C,D). Treatment with the PI3K inhibitor LY294002 (15 μmol/L) had no effect on BRIP1 expression but effectively reversed the increased p-PI3K/p-Akt levels induced by *BRIP1* overexpression (*p* < 0.05, Figure 6F, Scheme S6), positioning BRIP1 upstream of the PI3K/Akt pathway.

**Figure 7:**
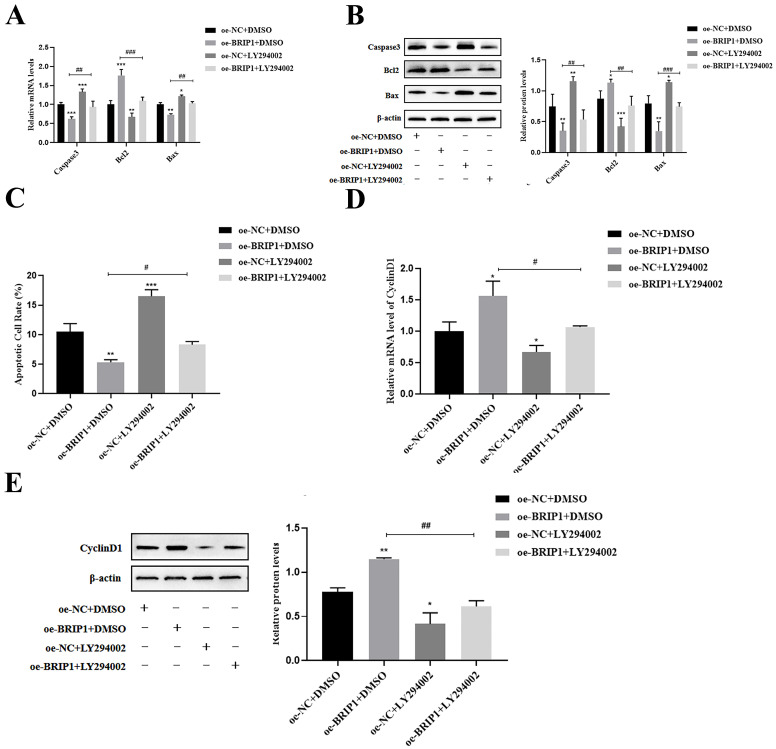
**BRIP1 regulates the anoikis resistance, cell cycle, and cell proliferation of GC via the PI3K/Akt pathway.** (**A**) Effect of LY294002 on mRNA expression in apoptosis-related genes. (**B**) Analysis of the effect of LY294002 on apoptotic protein. (**C**) Assessment of apoptosis rates by flow cytometry following LY294002 treatment. (**D**) The effect of LY294002 on *CyclinD1* mRNA was detected. (**E**) The effect of LY294002 on CyclinD1 protein was analyzed. * *p* < 0.05; ** *p* < 0.01; *** *p* < 0.001 compared to control; # *p* < 0.05; ## *p* < 0.01; ### *p* < 0.001.

**Figure 8:**
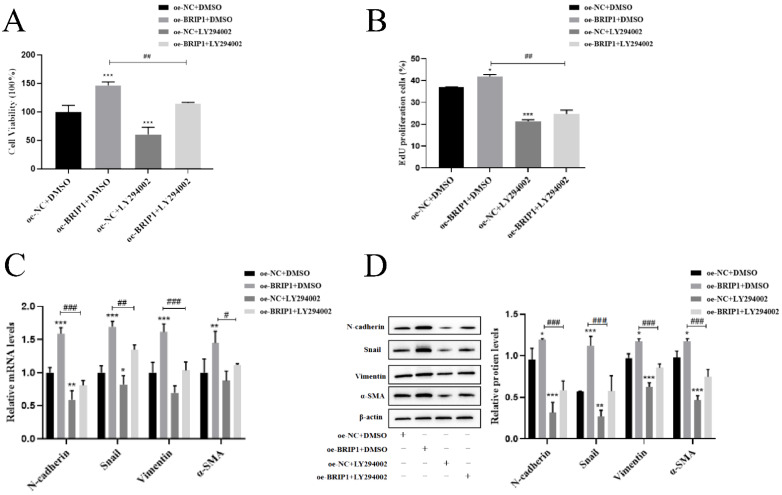
**BRIP1 regulates EMT process in GC through PI3K/Akt pathway activation.** (**A**) Cell viability assessment by CCK8. (**B**) EdU proliferation assessment rate. (**C**) mRNA expression levels of EMT-related markers (*N-cadherin*, *Snail*, *Vimentin*, *α-SMA*). (**D**) Protein expression levels of EMT-related markers. * *p* < 0.05; ** *p* < 0.01; *** *p* < 0.001 versus control; # *p* < 0.05; ## *p* < 0.01; ### *p* < 0.001.

We further verified that BRIP1 exerts its oncogenic functions through this pathway. LY294002 treatment reversed the anti-apoptotic effects of *BRIP1* overexpression, as evidenced by upregulated Caspase-3 and Bax, downregulated Bcl-2 levels (*p* < 0.05, Figure 7A,B), and a significantly elevated apoptosis rate in flow cytometry (*p* < 0.05, Figure 7C). Similarly, LY294002 suppressed BRIP1-driven cell cycle progression by downregulating CyclinD1 (*p* < 0.05, Figure 7D,E) and inhibited proliferation in CCK-8 and EdU assays (*p* < 0.05, Figure 8A,B). PI3K inhibition also significantly reduced the expression of EMT markers (*p* < 0.05, Figure 8C,D). These results show that BRIP1 acts upstream of the PI3K/Akt pathway, where it dampens ROS production and activates PI3K/Akt signaling to coordinately enhance anoikis resistance, cell cycle progression, proliferation, and EMT in GC.

## 3. Discussion

At present, the prevalence of GC in China remains at a relatively high level, with a relatively low five-year survival rate [3]. In recent years, many researchers have been seeking newer and more powerful therapeutic targets in order to prevent and treat GC more effectively, thereby improving patient survival and quality of life.

In this paper, we built a prognostic model for GC patients and found that *BRIP1* is related to the anoikis of GC cells. It confers anoikis resistance through concurrent ROS suppression and PI3K/AKT pathway activation. Cancer cells often resist anoikis, which is attributed to the alteration of the extracellular matrix and the disruption of intercellular interactions. This kind of disruption enables cancer cells to survive without relying on anchoring and promotes tumor growth, invasion and metastasis [22]. Anoikis resistance is a hallmark of metastatic progression and underlies tumor invasion and dissemination [9].

Here, through LASSO regression modeling, a prognostic risk assessment model composed of eight genes (*DUSP1*, *VCAN*, *P3H2*, *TXNIP*, *BRIP1*, *FGD6*, *GPX3*, and *RLN2*) related to anoikis was constructed. According to Wang et al., DUSP1 is a dual-specificity phosphatase [23]; it is essential for modulating ferroptosis in GC cells. Research by Huang et al. indicates that VCAN is not only associated with poor prognosis in GC but also correlates with immune infiltration, suggesting that it may be a key molecule in GC immunotherapy [24]. While research on P3H2 remains scarce, emerging evidence suggests its potential role as a breast cancer-specific cancer-inhibiting gene [25]. BRIP1, encoding a DNA helicase, was incorporated into a novel nine-gene prognostic signature for GC, which demonstrates robust predictive accuracy for patient outcomes and holds promise as a therapeutic target [26,27]. This signature integrates multiple genes with documented roles in GC pathogenesis: TXNIP is implicated in oxidative stress-related tumor progression [28]; FGD6 upregulation correlates with poor prognosis [29]; GPX3 overexpression suppresses migration and invasion [30]; and RLN2 has emerged as a promising candidate for drug development [31]. In our model, these genes collectively showed significant prognostic relevance. The model exhibited high predictive accuracy across both training and test datasets. Using the model’s risk score to categorize GC patients identified a striking difference in survival between high- and low-risk cohorts. This approach is supported by the growing application of machine learning in clinical decision-making [32]. Specifically, the LASSO algorithm—employed and validated here [33,34]—enhances model generalizability and interpretability. Our work thus builds upon prior efforts, such as the *BRIP1*-containing signature established by Wang et al. [27], to provide a clinically valuable tool for GC prognosis stratification. Here, we can apply the model constructed in this experiment to clinical practice and provide prognostic reference values for patients with GC.

BRIP1 has been widely implicated in the pathogenesis of multiple cancers, including ovarian [35], breast [36], colorectal [37], and GC [38,39]. Consistent with its reported roles, our functional experiments demonstrated that BRIP1 enhances proliferative capacity and promotes EMT in GC. These findings align with a previous report on triple-negative breast cancer, where BRIP1 loss led to upregulated expression of proliferation, stemness, angiogenesis, and metastasis markers, thereby enhancing invasiveness and tumor progression [40]. Furthermore, BRIP1 expression has been associated with the regulation of apoptosis and cell cycle in various tumors [26]. In this study, we report the novel finding that BRIP1 enhances anoikis resistance and induces cell cycle dysregulation in GC. Consistent with its oncogenic role, *BRIP1* overexpression notably accelerated tumor growth in a nude mouse xenograft model based on established methodologies [41]. Furthermore, BRIP1 increased the expression of key oncogenic proteins in the resulting tumors, validating its function in vivo. Collectively, our findings demonstrate that BRIP1 facilitates anoikis resistance in GC, thereby revealing a therapeutic vulnerability.

Studies have shown that the nuclear DNA repair protein OGG1 not only participates in the repair of nuclear DNA but may also translocate to mitochondria to facilitate the repair of mitochondrial DNA (mtDNA). If mtDNA damage is not promptly repaired, it can lead to mitochondrial dysfunction and subsequently increase the production of ROS [42]. This study shows that BRIP1 promotes cell anoikis by inhibiting the generation of ROS. Here, we hypothesize that the repair protein BRIP1 may also suppress ROS production through a similar mechanism. Substantial evidence indicates that ROS signaling is a central regulator of anoikis in multiple cancers. This is exemplified by gliotoxin-induced, ROS-mediated anoikis in colorectal cancer [43] and the sensitization to anoikis via V-ATPase inhibition, which acts through enhanced ROS production and protein misfolding [44]. Interestingly, Du et al. reported that NOX4-derived ROS promotes anoikis resistance in GC via EGFR upregulation [45], suggesting context-dependent roles for ROS in cancer progression that align with our findings. The PI3K/AKT signaling pathway has been increasingly recognized as a crucial regulator of anoikis in GC. Multiple studies support this connection: CEBPB-mediated SERPINE1 activation promotes anoikis resistance through PI3K/AKT and EMT pathway activation [46]; CAV-1 enhances anoikis resistance via Src-dependent EGFR-integrin β signaling and subsequent PI3K/AKT phosphorylation [47]; and hypoxia-induced ANGPTL4 activates the FAK/Src/PI3K-Akt axis to suppress anoikis [48]. Building on this evidence, our study provides the first demonstration that BRIP1 promotes both anoikis resistance and EMT in GC cells through PI3K/AKT pathway activation, although the complete signaling network requires further elucidation. While previous research has established ROS-mediated regulation of PI3K/Akt signaling in cancer development [49], our study did not experimentally validate the proposed “BRIP1-ROS-PI3K/Akt” axis, representing an important direction for future investigation.

Our research also has other shortcomings. First, the clinical sample size requires expansion to enhance the generalizability and robustness of our risk scoring model, ensuring its applicability to a broader patient population. Second, while our mouse model effectively captured key aspects of tumor proliferation, the inherent species differences necessitate cautious interpretation of results for clinical translation.

In conclusion, our research establishes *BRIP1* as a promoter of anoikis resistance in GC through ROS suppression and identifies its novel role in regulating both anoikis resistance and EMT via PI3K/AKT signaling. These findings offer a theoretical basis for and scientific evidence of *BRIP1* as a target for GC.

## 4. Materials and Methods

### 4.1. Patient Samples

Paired frozen specimens of GC tissue and matched normal tissue were sourced from 20 patients at Zhongda Hospital, affiliated with Southeast University. RNA and protein were extracted from these tissues for analysis by RT-qPCR and Western blot (WB), respectively. This work was formally approved by the hospital’s ethics committee. Following the procurement of informed consent from all patients, tissue specimens were collected in strict accordance with the ethical principles set forth in the Declaration of Helsinki. Tissue samples were taken by clinicians from patients with GC whose tumors had been surgically removed. Inclusion criteria: (1) pathological confirmation of GC; (2) no history of radiotherapy or chemotherapy prior to surgery; and (3) sufficient health to be eligible for a surgical procedure. Patients were excluded based on: (1) a concurrent diagnosis of other malignancies or digestive system diseases; (2) pre-operative assessment deeming them unsuitable for radical gastrectomy; or (3) perioperative mortality resulting from surgical complications.

### 4.2. Cell Culture

The GES-1 and HGC-27 cell lines were sourced from the Guangzhou Secu Biological Company (Guangzhou, China); AGS gastric adenocarcinoma cells were acquired from the Nanjing Wobixin Biological Company (Nanjing, China). All cell lines were maintained and cryopreserved at Southeast University.

### 4.3. Animal Studies

This paper utilized 3-week-old male BALB/c nude mice, supplied by Beijing Weitonglihua Animal Company (Beijing, China). All animal experiments were approved by the Animal Experiment Ethics Committee of Southeast University and conducted at its Animal Experiment Center. Following randomization, the mice were assigned to experimental groups of six animals each. Individual identification was achieved by ear notching. A week later, after the nude mice acclimated to the environment, they were ready for the experiments.

### 4.4. Bioinformatic Analysis

Transcriptomic data from GC tissue and matched normal tissue samples were obtained from the TCGA database. This data acquisition was conducted in full accordance with NIH TCGA Human Subject Protection and Data Access Policies; as such, no separate ethics committee approval was required for this study. ARGs were sourced from the gene sets available on the GSEA website. Key ARGs were identified by taking the overlap in a Venn diagram. Related data were sorted and analyzed by R Studio (4.4.1).

### 4.5. Construction and Evaluation of Prognostic Model

In this paper, a more robust risk scoring model was developed using LASSO regression. Cross-validation was performed using the glmnet R package. After 1000 rounds of cross-validation, the optimal LASSO regression lambda was identified, and then, the median risk score was obtained. Using the median risk score as a break point, we classified patients into low- and high-risk groups. Finally, appropriate ARGs were identified to develop the risk model. The score of this model is calculated by multiplying the coefficient obtained from the model by gene expression. Univariate and multivariate Cox regression analyses were performed to evaluate the independent prognostic value of the risk score alongside key clinicopathological variables. A predictive nomogram incorporating the risk score, age, sex, tumor grade, and stage was generated using the “rms” package. The calibration curve plotted with the “survival” package demonstrated good concordance between predicted and observed outcomes.

### 4.6. Stable Cell Lines Selection Assay

In order to confirm the appropriate infection conditions, the experiments were divided into the following categories according to different culture conditions: a blank control (RPMI-1640 complete medium only), a negative control (RPMI-1640 medium with HiTransG A) and a viral group (RPMI-1640 medium with HiTransG A and virus). Cells in the viral group were assigned to subgroups based on multiplicity of infection (MOI) values of 10, 20, 50, and 100. Each group has 3 parallel multiple holes. Cells were plated in 24-well plates at a density of 5 × 10^3^ cells per well and allowed to adhere for 24 h until they reached approximately 30% confluence. Equal amounts of RPMI-1640 complete culture medium and viral enhancement solution HiTransG A were added; the virus volume (in μL) required per well was calculated using the following formula: virus volume = [MOI × number of cells]/[viral titer (TU/mL)] × 10^3^. The cells were gently shaken and cultured in an incubator containing 5% carbon dioxide. Then, 16 h after infection, the cells were replaced with the complete RPMI-1640 medium and continued to be cultured for 72 h to observe cell transfection. The infection conditions for normal cell growth and optimal transfection efficiency were determined through pre-experiments as follows: the infection efficiency of AGS cells was determined by knocking down BRIP1 lentivirus (MOI = 50) and when overexpressing lentivirus (MOI = 80) reached 80%. We conducted subsequent experiments under this condition.

### 4.7. Western Blot Analysis

Cellular proteins were extracted using RIPA lysis buffer supplemented with a protease inhibitor cocktail (Yamei, Guangzhou, China). Protein concentrations were quantified with a BCA assay kit (Biyuntian, Shanghai, China). Equal amounts of protein were separated by 10% SDS-PAGE (Bio-Rad, Hercules, CA, USA) and subsequently transferred to a PVDF membrane (Millipore, Burlington, MA, USA). The membrane was blocked with 5% non-fat milk for 1 h at room temperature, followed by an overnight incubation with primary antibodies at 4 °C. After washing with TBST, the membrane was incubated with an HRP-conjugated secondary antibody for 1 h at room temperature. Protein bands were visualized using an enhanced chemiluminescence (ECL) detection system and imaged. All antibodies used are listed in Supplementary Table S1.

### 4.8. Real-Time Quantitative PCR Analysis

A suitable primer sequence was designed according to the requirements of the experiment, as shown in Supplementary Table S2. The extracted cDNA was configured with a reaction system in the enzyme-free EP tubes (Kangrun, Guangzhou, China) of an RT-qPCR kit (Nuoweizan, Nanjing, China), and a configured reaction system was pre-denatured at 95 °C. 5 min; cycle reactions (40 cycles) were performed at 95 °C for 5 min and 60 °C for 30 s. All reactions were performed in triplicate. Relative gene expression levels were determined using the 2^ΔΔCt^ method, with normalization to the GAPDH gene.

### 4.9. Apoptosis Assay

The apoptotic rates of adherent and suspended cell populations were determined with an Annexin V-FITC/PI kit (Yisheng, Beijing, China) following the manufacturer’s protocol. In brief, after washing with PBS, cells were subjected to a 15 min staining procedure with Annexin V-FITC and propidium iodide (PI) in the dark. Flow cytometric analysis was then performed on a Thermo instrument (Thermo, Waltham, MA, USA) within 1 h of staining.

### 4.10. Cell Cycle Analysis

Cells were rinsed with PBS pre-cooled at 4 °C, and then anhydrous ethanol pre-cooled at 4 °C was added to fix the cells overnight. On the second day, pre-cooled PBS at 4 °C was added, and then, 500 μL of staining solution was added at 37 °C for 30 min. The staining buffer was prepared by mixing the base dyeing buffer, 20 × PI staining solution, and 50 × RNase A at a volume ratio of 100:5:2. Cell cycle detection was performed using flow cytometry.

### 4.11. CCK-8 Assay

To assess proliferation, we used a CCK-8 kit (Biyuntian, China). GC cells were washed with PBS, resuspended in complete RPMI-1640 medium at 5 × 10^5^ cells/mL, and plated in 96-well plates (Corning, Kangrun, China). Following the treatment period, 10 µL of CCK-8 solution was added to each well, and the plates were incubated at 37 °C for 2 h. The absorbance at 450 nm was then measured using a microplate reader (TECAN, Männedorf, Switzerland) to determine proliferative activity.

### 4.12. EdU Assay

Proliferating cells were identified using a commercial EdU kit (Biyuntian, China). Following a 3 h pulse with 10 μM EdU at 37 °C, cells were fixed with 4% paraformaldehyde and permeabilized with 0.3% Triton X-100. The incorporated EdU was then stained by incubation with a Click-iT reaction cocktail for 30 min in the dark. After extensive washing, the samples were subjected to flow cytometric analysis.

### 4.13. Immunohistochemistry (IHC)

For IHC, slides were retrieved with EDTA-Citrate Antigen Retrieval Solution (Beyotime, Shanghai, China). Briefly, 1% hydrogen peroxide was used to block the endogenous peroxidase activity. The slides were incubated with primary antibodies—anti-Caspase3, anti-Bcl2, anti-Bax, anti-CyclinD1, anti-Ki67, anti-N-cadherin, anti-Snail, anti-Vimentin, and anti-α-SMA—overnight at 4 °C and then incubated with secondary antibodies conjugated to peroxidase-labeled dextran polymer according to the manufacturer’s instructions. A light microscope was used to observe the slides. All IHC results were read blindly by two independent researchers.

### 4.14. Establishing a Tumor Formation Model of Nude Mice

To establish a xenograft model, a 200 μL cell suspension was injected subcutaneously into the left flank of each mouse. Tumor dimensions were recorded twice weekly using a caliper, and volumes were determined as V = (L × W^2^)/2. The mice were euthanized after two weeks, and the tumors were harvested and weighed. For downstream applications, tumor tissues were processed either by fixation in 4% paraformaldehyde for immunohistochemistry (IHC) or by snap-freezing for subsequent RNA extraction and RT-qPCR. Relevant primer sequences can be located in Supplementary Table S2.

### 4.15. Statistical Analysis

All statistical analyses and data visualization were conducted using R (v4.4.1), GraphPad Prism (v10), SPSS (v31), and Excel. Survival outcomes between groups were compared using the Kaplan–Meier method, and differences were assessed with the log-rank test. The prognostic independence of the risk model was evaluated using both univariate and multivariate Cox proportional hazard regression models. Differences in gene expression between groups were analyzed using a two-tailed Student’s *t*-test. A two-sided *p* < 0.05 was considered statistically significant. All data are presented as the mean ± SD unless otherwise stated.

## 5. Conclusions

This study demonstrates that BRIP1 orchestrates anoikis resistance in gastric carcinogenesis through dual mechanisms of ROS dampening and PI3K/Akt pathway potentiation. This work fundamentally advances our understanding of GC pathophysiology by characterizing BRIP1’s novel role, simultaneously revealing its utility as a clinical biomarker and exposing it as a target for therapeutic intervention.

## Data Availability

The data that support the findings of this study are available from the corresponding author upon reasonable request.

## Supplementary Materials

The following supporting information can be downloaded at https://www.mdpi.com/article/10.3390/ijms27052409/s1.

## Abbreviations

GC	Gastric cancer
EMT	Epithelial–mesenchymal transition
IARC	International Agency for Research on Cancer
ARGs	Anoikis-related genes
TCGA	The Cancer Genome Atlas
GSEA	Gene Set Enrichment Analysis
LASSO	Least Absolute Shrinkage and Selection Operator
RT-qPCR	Reverse transcription quantitative polymerase chain reaction
cDNA	Complementary DNA
DEPC	Diethyl pyrocarbonate
FBS	Fetal bovine serum
PI	Propidium iodide
PBS	Phosphate buffer saline
DMSO	Dimethyl sulfoxide
DEGs	Differentially expressed genes
ECM	Extracellular matrix
WB	Western blot
PAGE	Polyacrylamide gel electrophoresis
PVDF	Polyvinylidene fluoride
PMSF	Phenylmethanesulfonyl fluoride
CCK-8	Cell counting kit-8
EdU	5-Ethynyl-2′-deoxyuridine
IHC	Immunohistochemistry
ROS	Reactive oxygen species
TBST	Tris buffer saline Tween-20
MOI	Multiplicity of infection
