# Supplementary material for: Role and Mechanism of BRIP1 in Anoikis Resistance of Gastric Cancer
Source: Int J Mol Sci. 2026 Mar 5;27(5):2409. doi: 10.3390/ijms27052409 (PMC12985691; doi:10.3390/ijms27052409)
Supplement: Supplementary file 1 [file ijms-27-02409-s001.zip › Supplementary Schemes.pdf]

International Journal of
Molecular Sciences

MDPI

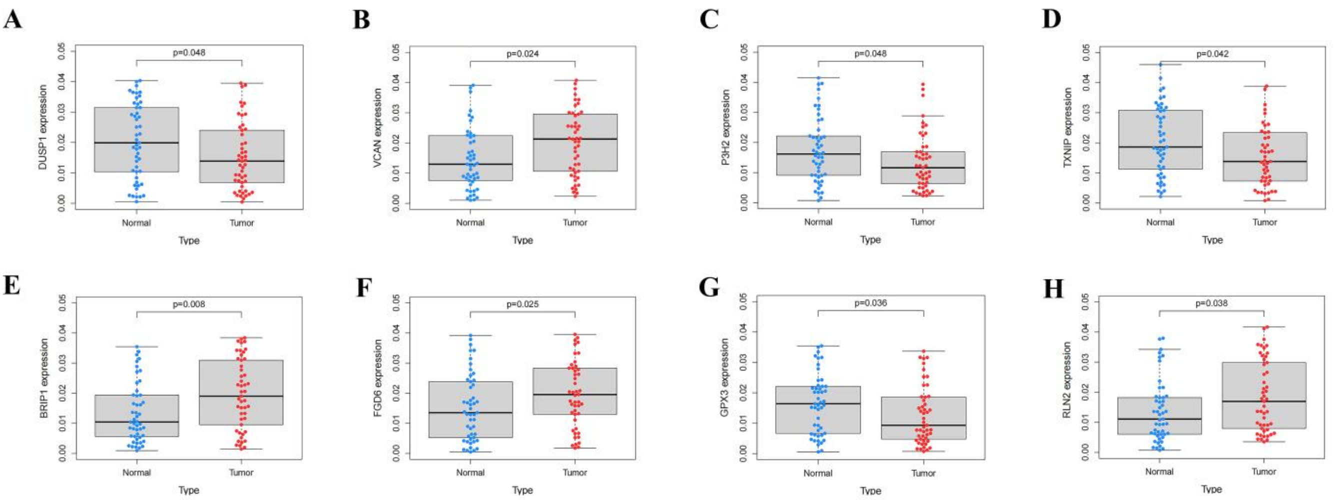

Scheme S1. The validation of ARGs in tissue samples. A Downregulation of DUSP1 in tumors. B Upregulation of VCAN in tumors. C-D P3H2 (C) and TXNIP (D) are significantly downregulated in GC tissues. E-F BRIP1 (E) and FGD6 (F) are significantly upregulated in GC tissues. G Downregulation of GPX3 in GC tissues. H Upregulation of RLN24 in tumors.

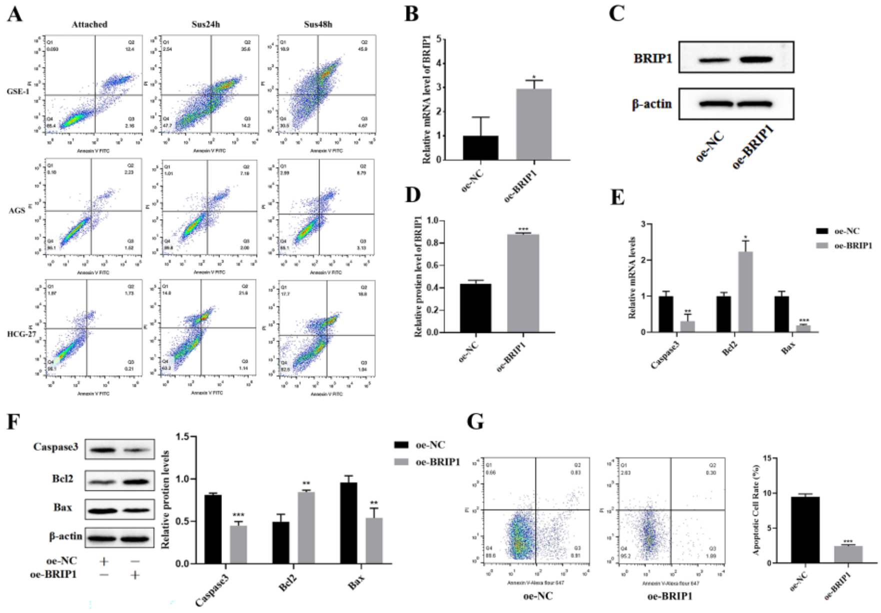

Scheme S2. A Apoptotic rates were assessed in GES-1, AGS and HGC-27 after 24 and 48 hours of adherent or suspension culture by flow plot. B-D Efficiency of upregulated *BRIP1* was detected. (B) represents RNA levels, while (C) and (D) represent protein levels. E Analyzed the mRNA expression of apoptosis-related genes. F The expression of apoptosis-related proteins was assessed. G Analyzed the effect of BRIP1 on apoptosis rate. $*P < 0.05$; $**P < 0.01$; $***P < 0.001$.

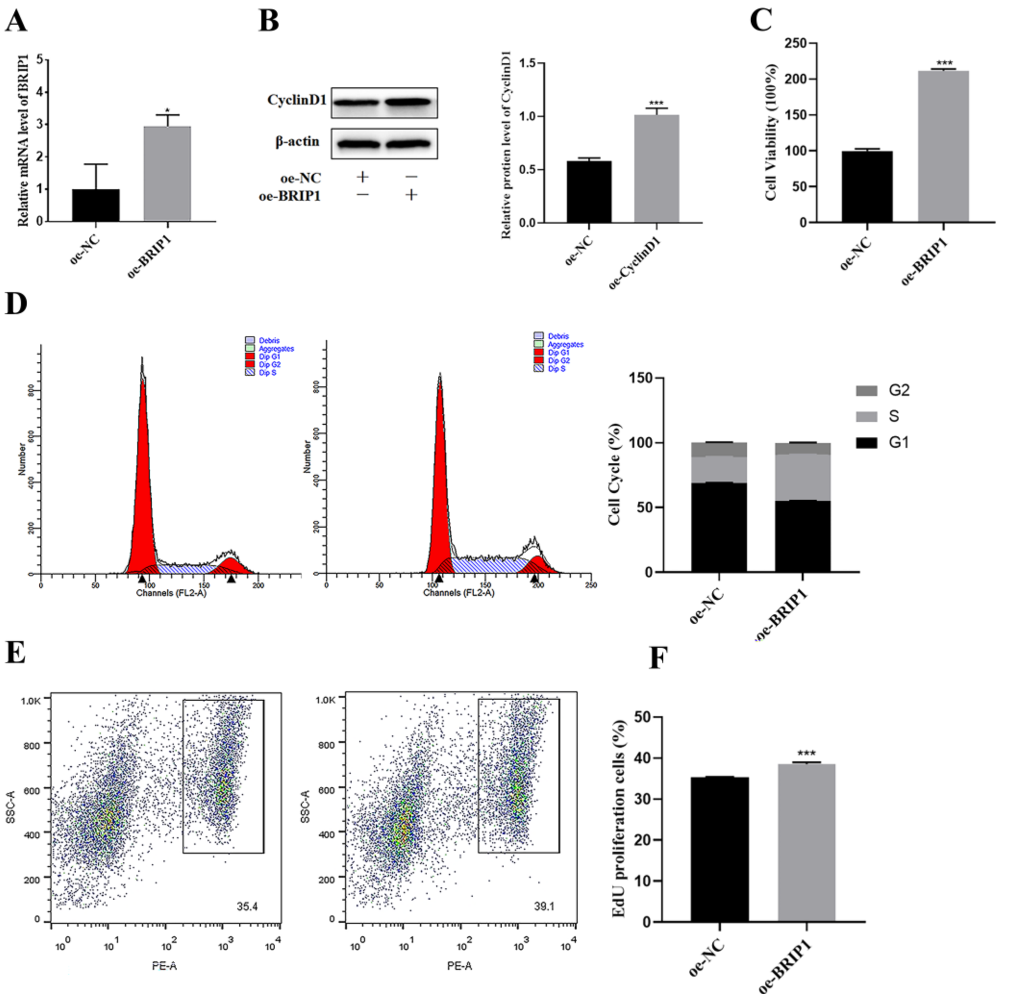

Scheme S3. **A** *BRIP1* regulated the expression of *CyclinD1* mRNA. **B** WB analysis of CyclinD1 protein expression regulated by *BRIP1*. **C** Cell cycle distribution analyzed by flow cytometry following *BRIP1* modulation. **D** Assessment of cell viability by CCK-8 assay. **E-F** Cell proliferation assessed by EdU assay.

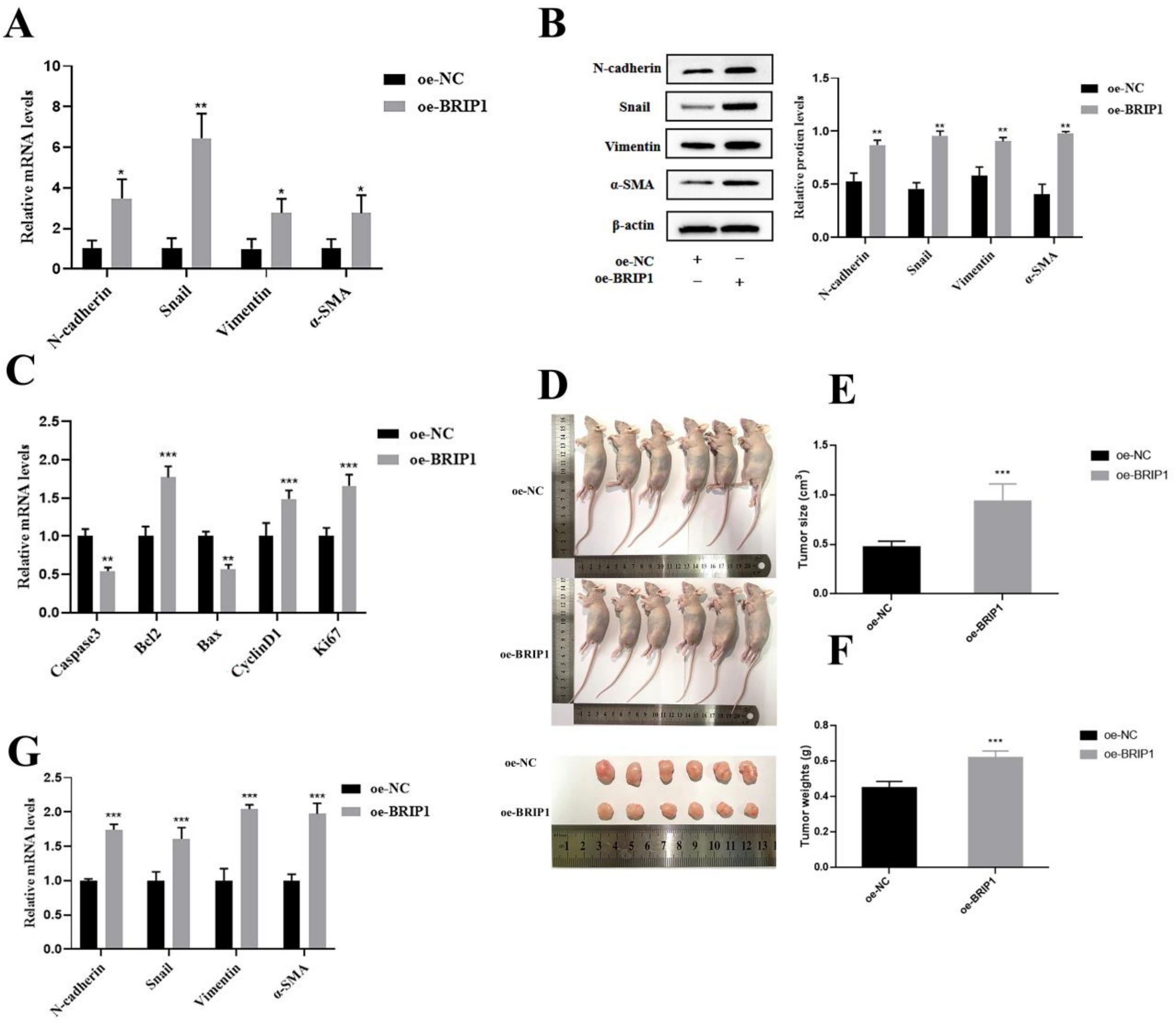

Scheme S4. **A** The mRNA expression of EMT gene regulated by *BRIP1* was detected. **B** Analyzed the expression of EMT protein regulated by *BRIP1*. **C** Assessed the expression of apoptosis, cycle and proliferation genes in tumor tissues. D In vivo tumor formation experiment with overexpression of *BRIP1*. **E-F** Tumor size and weight in the overexpression group. **G** Expression of EMT-related genes in tumor tissues. *$P < 0.05$; **$P < 0.01$; ***$P < 0.001$.

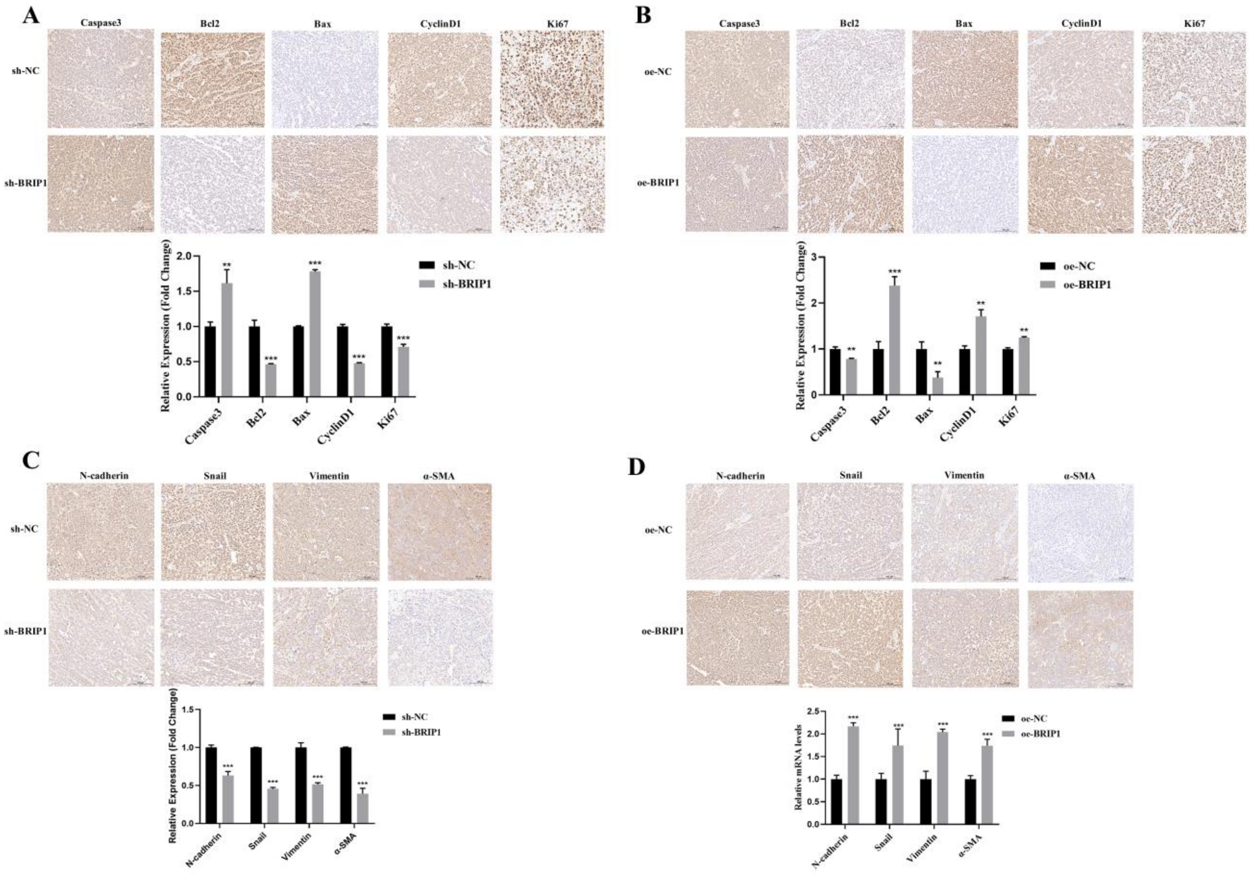

Scheme S5. BRIP1 affects protein expression related to apoptosis, proliferation, and EMT in nude mouse axillary tumors. A-B IHC analysis of apoptosis- (Caspase-3, Bax, Bcl-2), cell cycle- (Cyclin D1), and proliferation-related (Ki67) proteins in axillary tumor tissues from nude mice after *BRIP1* knockdown or overexpression. **C-D** IHC evaluation of EMT-related proteins (N-cadherin, Snail, Vimentin, α-SMA) in the same tumor models. *$P < 0.05$; **$P < 0.01$; ***$P < 0.001$.

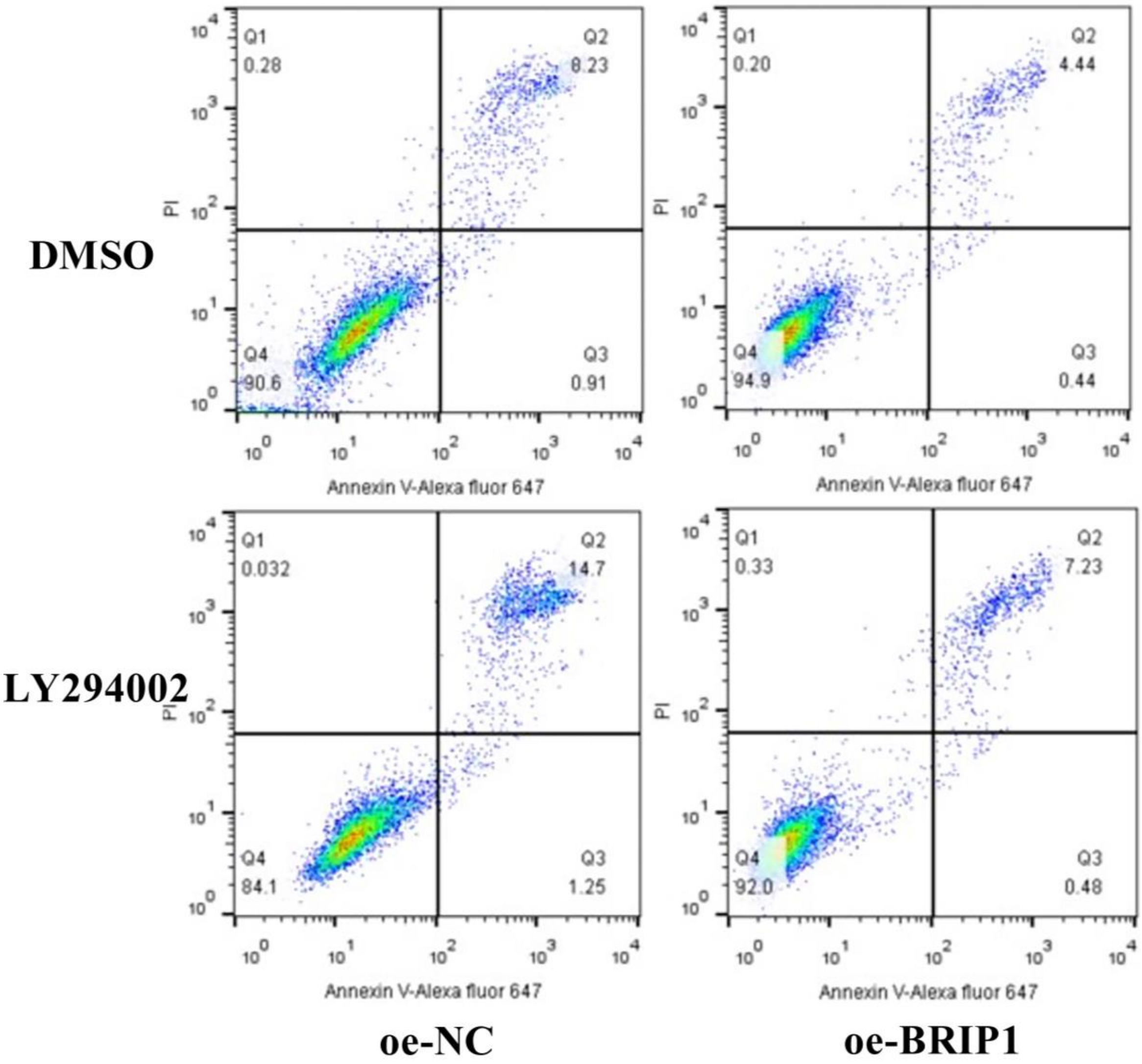

Scheme S6. A Assessment of apoptosis rates by flow cytometry following LY294002 treatment.
