# Supplementary material for: Role and Mechanism of BRIP1 in Anoikis Resistance of Gastric Cancer
Source: Int J Mol Sci. 2026 Mar 5;27(5):2409. doi: 10.3390/ijms27052409 (PMC12985691; doi:10.3390/ijms27052409)
Supplement: Supplementary file 1 [file ijms-27-02409-s001.zip › Supplementary Table S1.pdf]

Supplementary Table S1 Experimental antibody

Name	Source
Primary antibody β-actin	Sanying, China
Primary antibody BRIP1	Sanying, China
Primary antibody Snail	Aibotaike, China
Primary antibody Caspase3	Aibotaike, China
Primary antibody Bcl2	Aibotaike, China
Primary antibody BAX	Aibotaike, China
Primary antibody CyclinD1	Aibotaike, China
Primary antibody α-SMA	Aibotaike, China
Primary antibody N-cadherin	Aibotaike, China
Primary antibody Vimentin	Aibotaike, China
HRP-conjugated goat anti-rabbit secondary antibody	Huaan, China
HRP-conjugated goat anti-mouse secondary antibody	Huaan, China
Primary antibody AKT	Sanying, China
Primary antibody p-AKT	Sanying, China
Primary antibody PI3K	Sanying, China
Primary antibody p-PI3K	Sanying, China
