# Supplementary material for: Role and Mechanism of BRIP1 in Anoikis Resistance of Gastric Cancer
Source: Int J Mol Sci. 2026 Mar 5;27(5):2409. doi: 10.3390/ijms27052409 (PMC12985691; doi:10.3390/ijms27052409)
Supplement: Supplementary file 1 [file ijms-27-02409-s001.zip › Supplementary Table S2.pdf]

Supplementary Table S2 Primer sequence

Name	Sequence
VCAN (Human)	F: CCATCTCACAAGCATCCTGTCTCAC<br>R: CTGCCATCAGTCCAACGGAAGTC
FEN1 (Human)	F: TGGTGAAGGCTGGCAAAGTCTATG<br>R: CTGGCAGTCAGGTGTCGCATTAG
BRIP1 (Human)	F: GAAACCAGCAGATGAGGGCGTAAG<br>R: AGAAGGTGGTGTGCTTGGATAGTTG
CNTN1 (Human)	F: AAGCACTGAAGCAACCCTGAGC<br>R: TCACAGAGAAGCACCATTCCTTTCC
P3H2 (Human)	F: GTGTGCTGTGGCTCTGTGGTTC<br>R: TGCTTCCCTTGCTGTTCTTGATCC
DUSP1 (Human)	F: GGATACGAAGCGTTTTCGGC<br>R: AGAGGTCGTAATGGGGCTCT
GAPDH (Human)	F: TCAAGATCATTGCTCCTCCTGAG<br>R: ACATCTGCTGGAAGGTGGACA
Caspase3 (Mouse)	F: GGGACTGATGAGGAGATGGCTTG<br>R: AAAGGGACTGGATGAACCACGAC
Bcl2 (Mouse)	F: CTACGAGTGGGATGCTGGAGATG<br>R: GGTTGCTCTCAGGCTGGAAGG
BAX (Mouse)	F: CATGGGCTGGACACTGGACTTC<br>R: AGCGAGGCGGTGAGGACTC
CyclinD1 (Mouse)	F: AGGCGGATGAGAACAAGCAGAC<br>R: TGGAGGGTGGGTTGGAAATGAAC
Ki67 (Mouse)	F: CGCAGATCAGGCAGGACTTCC<br>R: CAGGCAGGTTACCGCTCTTCC
N-cadherin (Mouse)	F: AGCTCCCTCAACTCCTCCAGTAG<br>R: GTCGTCACCACCGCCGTAC
Snail (Mouse)	F: AAGACGACATAGACGGCATCCAG<br>R: TCGGCTGTGGTTCAGTTGTGG
Vimentin (Mouse)	F: CACTAGCCGCAGCCTCTATTCC<br>R: AAGTCCACCGAGTCTTGAAGCAG
α-SMA (Mouse)	F: GGATGAAGCCCAGAGCAAGAGAG<br>R: TGTCGTCCCAGTTGGTGATGATG
GAPDH (Mouse)	F: GCAAATTCAACGGCACAGTCAAG<br>R: TCGCTCCTGGAAGATGGTGATG
Caspase3 (Human)	F: GTGGAGGCCGACTTCTTGTATGC<br>R: TGGCACAAAGCGACTGGATGAAC
Bcl2 (Human)	F: GCCAGCACCATATCCATTCGTCAG<br>R: ACTCCACAGCGACAGCAAAGAAC
BAX (Human)	F: GGGGACGAACTGGACAGTAA<br>R: CAGTTGAAGTTGCCGTCAGA
CyclinD1 (Human)	F: AAACAGATCATCCGCAAACAC<br>R: GTTGGGGCTCCTCAGGTTC

N-cadherin (Human)	F: CGGTGCCATCATTGCCATCCT<br>R: GTCATAGTCCTGGTCTTCTTCTCC
Snail (Human)	F: AAGGGTACAGCCTGTTCCTGGT<br>R: CTGGATGCCGTCTATGTCGTCT
Vimentin (Human)	F: GGTTCAGGTTTCATTCATGCCT<br>R: AGTTGGCTGTGTGTACTGCT:
α-SMA (Human)	F: AGACGGGAATCCTGTGAAGC<br>R: TGTCCCATTCCCACCATCAC
